# American Educational Journalism

**Published:** 1889-03

**Authors:** 


### CULLINGS FROM CONTEMPORARIES.

### AMERICAN EDUCATIONAL JOURNALISM.

Dr. T. C. Minor, in a recent number of the Cincinnati Lancet-Clinic, relieves his mind in the following strong, if not elegant, language:

"For several months past the leading journals of civilization (?) in the country, *i. e.*, Harper's and the Century, have devoted much of their advertising space to patent medicines and quack professional cards. As the exponents of the culture they represent and the educational purposes they serve, their advertisements, it may be taken for granted, afford a correct index as to the intelligence of their readers, who, it is safe to presume, are governed by the medical advice of the able editors.

"Among the numerous nostrums, largely billed and illustrated, may be taken, as an example, a preparation known as 'Recamier

Cream,' a thing that Adelina Patti Nicolini—she of two or three husband fame—and other women of similar moral character, cry after; even that much-manned old French frigate, Sarah Bernhardt, weeps for joy when she pastes this delightful bichloride preparation on her pimpled cheek and fires off a broadside of bad French eulogy.

"It was Rasselas, prince of Abyssinia, who exclaimed: 'Ye who listen with credulity to the whispers of fancy, and pursue with eagerness the phantoms of hope, who expect that age will perform the promises of youth and the deficiencies of the present day will be supplied by to-morrow, listen to the story of Rasselas.' This story may be found fully set forth in the advertising of 'Recamier's Cream' and a preparation known as 'Vita Nuova;' for artistic lying the writer of these cards could give Ananias points and then treble discount him. In order to do this, however, it is necessary to invoke the aid of the popular actress of ill-repute and the talented preacher of God's holy word—a strange combination, forsooth, but one that always hits that most easily gulled of all human beings, the so-called *bright* American, the principal patron and worshiper of humbuggery—for in America religion and the stage, with patent medicine, wander together hand in hand, seeking to delude the dear people, who are a fair prey for the average impostor. Pick up any religious or temperance paper in the country, and there you will find 'Bitters' that contain alcohol, and the 'Opium Antidote' that is saturated with morphine. Without such 'ads.' theological journals would not thrive in the United States; and this tendency to perpetuate fraud through unscrupulous jornalism has now extended like a pestilence to the lay journals of the land. The mischief wrought by these foul destroyers of soul, mind and body is incalculable; they are corruptors of morality, the insidious iconoclasts of public virtue, and the paid agents of vice; the price of the 'ad.' soothes each drowsy conscience in a land where the struggle is for wealth, no matter how close the victim grazes the penitentiary bars in the pursuit of gain. The religious journals have for years been panderers to the venders of abortive remedies; Christ is crucified in one column and pennyroyal and cotton-wood pills praised on the opposite page. It is no wonder that physicians, year by year, are evidencing a wider tendency to denounce religious and so-called moral journalism. The most sensational morning journal in the country would modestly shrink from publishing the filthy 'ads.' found in some of the religious weeklies of the United States, where the 'retired clergyman, rnined by early indiscretions,' etc., publishes his card with the holy address of 'Bible House,' New York.

"It seems to be a popular belief that the regular medical profession objects to patent medicine because it interferes with their practice; such is not the case, for these nostrums are largely responsible for the Bright's disease and bladder troubles of this country. Every dozen bottles of patent medicine sold over the druggist's counter makes a patient for the doctor. It is not difficult to cure disease oftentimes, but the present epidemic of patent medicine damphoolery, nurtured and fostered in the interests of the various churches of America, should be restrained. If clergymen desire respect for their calling, they should preach what they practice. The 'Bitters' in the study closet, while an aid to preparing the usual dull Sunday sermon, have enough alcohol in them to induce the clerical cirrhosis of the liver, or theological brain softening, which seems to be a common complaint just at the present period."

---

THERE'S A GOOD TIME A COMING.—The City council of San Antonio have appropriated $500 to aid the local committee of arrangements in providing entertainment for the Texas State Medical Association, which will meet in that city on 23d prox. In addition, the citizens have subscribed liberally, and we understand a large fund has been raised. There will doubtless be a "feast" of of other things beside that of "reason," and another "flow" beside the "flow of soul." "Mum's" the word!

---

TAXING DENTISTS.—Another outrageous law disgraces our statute books, and should be repealed, the law requiring dentists to pay an occupation tax. Dentists are doctors, and the occupation tax on physicians has been repealed.

